# Bacteria as Therapeutic Agents

**Published:** 1885-10-20

**Authors:** 


### BACTERIA AS THERAPEUTIC AGENTS.

It is a fact repeatedly demonstrated in the experience of those engaged in culture-experiments that some of the recognized pathogenic schizomycetes are very liable to destruction by the ordinary bacteria of putrefaction. The suggestion has been half humorously, half seriously made that this might be utilized in the treatment of disease, but the honor of the first actual application of this physiological antagonism to therapeutics appears to belong to Prof. Arnoldo Cantani, of Naples.* The first experiments made by Prof. Cantani were directed to tuberculosis, and he selected the bacterium termo to antagonize the bacillus tuberculosis of Koch. He proceeded first to determine the effects of bacterium termo on animal organism when applied to the conjunctiva, or injected into the stomach or under the skin, and especially when inhaled into the lungs in the form of spray. The results of numerous experiments made upon animals demonstrated conclusively the complete innocuousness of this form of bacteria when introduced in this way. He then administered a solution of a pure culture of the bacterium termo to a patient forty-two years of age, presenting the usual symptoms of acute pulmonary phthisis—fever, great emaciation, and loss of strength—with a cavernous excavation in the left apex, and sputa containing tubercle-bacilli in abundance. The results were sufficiently striking to give in his own words: "On the 4th of May the inhalations began, using a pure culture of bacterium termo diluted with gelatin and meatbroth, by means of a hand spray apparutus with double rubber bulb. The expectoration rapidly decreased, until it ceased entirely during the last few days of the experiment. The tubercle-bacilli likewise soon diminished from the expectoration, while the bacterium termo could now be detected in their place. On June 1 the tubercle-bacilli were completely absent from the expectoration, and did not reappear, while bacterium termo became more and more abundant (the expectoration being examined daily). The fever decreased so that during the last few days of the observation the evening temperature did not go above 38°C. (100.4°F.), and the bodily weight was decidedly increased. The appearance of the patient greatly improved, the general condition became excellent, and the patient felt much better." Although animals inoculated with the sputum at the beginning of the treatment had presented signs of local and afterwards of general tuberculosis, those inoculated with the sputa found to be free from tubercle-bacilli remained healthy and gave no trace of tubercular disease.

This demonstration satisfies the requirements of science, and apparently establishes it as a fact that the antagonism known to exist between the bacterium termo and the bacillus tuberculosis may be utilized for therapeutic purposes *cito, tuto. et jucunde.* At the same time, Prof. Cantani is not as sanguine as to the further application of this principle in treating cases where the bacilli have become embedded in remote organs ; but it affords a hope that something may be expected from this treatment where the tuberculous process in the lungs is not extensive and appears to be more or less superficial. It is also suggestive as regards the employment of other bacteria which are known to affect unfavorably the growth of certain pathogenic micro organisms.—*Medical Times.*

